# Anaphylaxis due to I.V. prednisolone application in two patients suffering from multiple sclerosis

**DOI:** 10.1186/2045-7022-4-S3-P8

**Published:** 2014-07-18

**Authors:** Tamar Kinaciyan

**Affiliations:** 1 Division of Immunology, Allergy and Infectious Diseases, Department of Dermatology, Medical University of Vienna, Austria

## Background

Just a few weeks apart, two women in their forties were presented by neurologists at our outpatient clinic. Both suffered from multiple sclerosis (MS) and were always successfully treated with i.v. prednisolone when in acute relapse. Both patients developed at the last treatment with prednisolone generalized urticara, dyspnea and hypotension. They were then treated and stabilized with antihistamines and volume expanders.

## Methods

Skin-Prick (SPT) and intradermal (i.d.) tests were performed with prednisolone as the probable causative agent and as alternatives with methylprednisolone, triamcinolone and dexamethasone. Additionally, drug provocation tests (DPT) with alternatives and / or SPT negative preparations were performed intravenously.

## Results

All prick tests resulted in both patients negative. In one patient, only i.d. test with prednisolone turned positive, all other steroids were negative and she tolerated i.v. application of negative tested steroids. In the other patient, the i.d. test with prednisolone and methylprednisolone were positive and she tolerated DPT with triamcinolone well.

## Conclusions

Corticosteroid preparations are used to treat allergic reactions and anaphylaxis but in patients who are often treated with systemic steroids, as it was the case in both of our patients with MS, allergic sensitization to steroids can develop. Therefore, allergologic work-up is required for all suspected corticosteroid immediate type reactions. As methylprednisolone may cross-react with prednisolone in some cases, in acute emergencies, it is recommended to switch to triamcinolone or dexamethasone.

